# Accounting for Linear Transformations of EEG and MEG Data in Source Analysis

**DOI:** 10.1371/journal.pone.0121048

**Published:** 2015-04-02

**Authors:** Joerg F. Hipp, Markus Siegel

**Affiliations:** 1 Centre for Integrative Neuroscience, University of Tübingen, Tübingen, Germany; 2 MEG-Center, University of Tübingen, Tübingen, Germany; University of Minnesota, UNITED STATES

## Abstract

Analyses of electro- and magnetoencephalography (EEG, MEG) data often involve a linear modification of signals at the sensor level. Examples include re-referencing of the EEG, computation of synthetic gradiometer in MEG, or the removal of artifactual independent components to clean EEG and MEG data. A question of practical relevance is, if such modifications must be accounted for by adapting the physical forward model (leadfield) before subsequent source analysis. Here, we show that two scenarios need to be differentiated. In the first scenario, which corresponds to re-referencing the EEG and synthetic gradiometer computation in MEG, the leadfield must be adapted before source analysis. In the second scenario, which corresponds to removing artifactual components to ‘clean’ the data, the leadfield must not be changed. We demonstrate and discuss the consequences of wrongly modifying the leadfield in the latter case for an example. Future EEG and MEG studies employing source analyses should carefully consider whether and, if so, how the leadfield must be modified as explicated here.

## Introduction

Analyses of EEG and MEG data often comprise two successive analysis steps. The first step involves linear transformations of the data at the sensor level. These transformations include re-referencing the EEG [1] or computing synthetic gradiometers in MEG [2], and cleaning procedures that aim to remove artifactual signal components from the data. Commonly independent component analysis (ICA) [3–6] is used for artifact cleaning [7–13]. In a second step, source analysis is applied to estimate the neuronal sources underlying the measured electrical potentials or magnetic fields [14]. The starting point for source analysis is the physical forward model, or ‘leadfield’, that describes the relation between electrical sources and sensors. A linear transformation of the sensor-level data before source analysis raises the question whether and, if so, how such linear transformation needs to be taken into account for source analysis. In particular, the question is, if the leadfield must be adapted to account for the altered representation of the data. This topic has been addressed for specific cases including signal space projection of MEG data [15] and computing the surface Laplacian of EEG data [16], or may be considered well-known for the case of EEG re-referencing [1,14,17]. However, a systematic and general consideration of this problem and practical guidelines are missing. Here we fill this gap.

## Material and Methods

### Participants, stimulus, and task

The third part of this paper presents a re-analysis of EEG data reported previously [8,18]. The study was conducted in accordance with the Declaration of Helsinki, approved by the local ethics committee (Ethics-Committee of the Medical Association of Hamburg), and written informed consent was obtained from all subjects prior to the recordings. Subjects (n = 20) fixated a central cross while two moving bars approached each other, overlapped, and diverged again (onset at 0 s, total duration, 1.52 s, size of bars 5° x 0.125° visual angle, starting position at 3.8° eccentricity, velocity: 5°/sec). A click-sound (duration: 20 ms, volume: 60 dB SPL) was played at the moment of bar overlap via a central loudspeaker. Subjects reported their percept of the stimulation (bouncing or passing bars) via button-press (left and right thumb) after fixation-cross offset on each for 500 trials.

### Data acquisition and preprocessing

We recorded the EEG from 126 scalp sites (sampling rate: 1000 Hz; high-pass: 0.01 Hz; low-pass: 250 Hz; Amplifier: BrainAmp, BrainProducts, Munich, Germany; Ag/AgCl ring electrodes mounted on an elastic cap, Falk Minow Services, Herrsching, Germany; nose reference). Electrode impedances were kept below 20 kΩ. Offline, the data were re-referenced to average reference, high-pass filtered at 4 Hz, and cut into trials of 2.5 s duration centered on the presentation of the sound. Trials with eye movements, eye blinks, or strong muscle activity were identified by visual inspection and rejected from further analysis. We employed independent component analysis (FastICA) [5] to remove artifactual signal components [8–13,19].

### Spectral analysis

We performed spectral analysis at 70 Hz in a sliding-window (250 ms window length; 19 time points from -0.14 to 1.66 s in 100 ms steps; tapers: discrete prolate spheroidal (slepian) sequences [20,21] using 12 tapers, corresponding to 1 octave spectral smoothing). The spectral estimate at -0.14 s served as the pre-stimulus baseline. For illustration the power time-courses were interpolated (shape-preserving piecewise cubic interpolation).

### Source analysis

We used adaptive linear spatial filtering (‘beamforming’) [22,23] to estimate the spectral power of neural population signals at the cortical source level. In short, for each time, frequency, and source location, 3 orthogonal filters (one for each spatial dimension) were computed that pass activity from the location of interest with unit gain, while maximally suppressing all other sources. The filters were computed separately for each point in time and frequency based on the real part of the cross-spectral density matrix of the data after subtraction of the event-related potential from each single trial. We linearly combined the 3 filters to a single filter in the direction of maximum variance.

**Fig 1:**
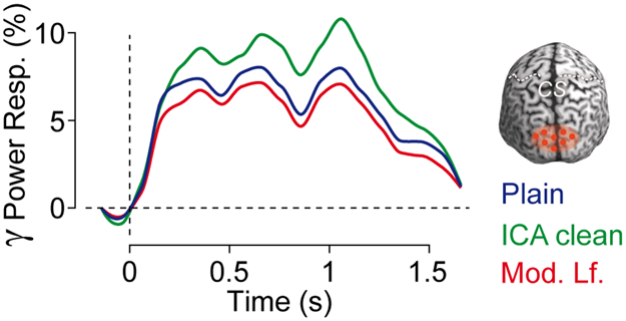
Effect of wrongly correcting the leadfield for source analysis following ICA cleaning. Time courses of source-level gamma band activity (50–100 Hz) in response to a moving visual stimulus (onset: t = 0 s; offset: t = 1.52 s) in an occipito-parietal region of interest (see top right). Plain: No ICA-based artifact cleaning. ICA clean: Artifactual ICA components are removed from the sensor level data. Mod. Lf.: As for ICA clean but additionally the leadfield was modified by mistake.

We reconstructed neuronal activity from 8 locations in the visual cortex (center MNI coordinate: [0–87 26], maximal distance from center: 21 mm, see Fig. 1) and averaged the power time-courses for illustration in Fig. 1. To derive the leadfield (physical forward model), we constructed a 3-compartment boundary element head-model from the segmented SPM99/2 template brain (skin; skull; brain including white matter, gray matter and cerebral spinal fluid; conductivities: 0.33, 0.041, 0.33 S/m, respectively) and co-registered average electrode positions. Finally, we transformed this generic head-model into the subjects’ individual head-space based on individual T1-weigthted structural magnetic resonance images (MRI) to derive individual leadfields. Head-model and leadfield construction was performed using Fieldtrip [24] and SPM (http://www.fil.ion.ucl.ac.uk/spm/).

## Results

The results are organized into 3 sections. First, we give an intuitive explanation of why the leadfield must be changed for re-referencing procedures and synthetic gradiometers, but must not be changed for cleaning procedures such as ICA cleaning. Then, we provide a formal explanation and detail how to account for changes of the sensor level data in the leadfield when necessary. Finally, we test and discuss in a real-world example what happens, if, following ICA cleaning of EEG data, the leadfield is corrected by mistake for source analysis.

### 1. Intuitive explanation

The leadfield is a physical model of the signal that would be measured by the sensors for a unit dipole with known location and orientation. In the case of re-referencing the EEG or computing synthetic gradiometer in MEG, the data is linearly transformed into a new set of signals reflecting measurements from a new set of virtual sensors. Consequently, the leadfields for the new representation of the data must be adapted [14]. In contrast, in the case of ICA cleaning, the physical meaning of the signals and the corresponding sensors is not changed, but rather a part of the signal is removed that was identified as an artifactual non-brain signal. Consequently, in this case, the leadfield must not be changed.

### 2. Formal description

#### Linear transformations of sensor level data

The first step in EEG or MEG analyses often involves linear transformations of the sensor level data of the general form:

$$\tilde{X}=WX$$ (1)

Where *X* and $\tilde{X}$ are matrices (channels x time points) representing the measured and transformed data, respectively, and *W* is the transformation matrix. The exact form of *W* depends on the linear transformation employed. Two common cases that we discuss next are re-referencing and ICA-based artifact cleaning.

#### Re-referencing

For re-referencing, *W* is the difference of the identity and a reference defining matrix *R*.

$$W_{ref}=I-R$$ (2)

*R* typically derives the average of all sensors (all elements are set to the reciprocal number of sensors) or selects a specific sensor (one column of ones). Similarly, transformation matrices *W* can be derived for related operations, such as e.g. computing the surface Laplacian of EEG data [1,16] or computing synthetic gradiometers in MEG [2].

#### ICA-based artifact cleaning

To derive the linear transformation matrix *W* for the case of ICA cleaning, we start with the ICA model and express the data as a weighted set of independent components *Y* (component x time points) realized by a mixing matrix *A* (channels x components) [5]:

X=AY (3)

In many real-world applications *A* and *Y* are unknown and are estimated from the data using e.g. independent component analysis (ICA) [3–6]. If the signals in *X* are independent, *A* is full rank, then *A* ^*-1*^ exists and *Y* can be derived as:

$$Y=A^{-1}X$$ (4)

Removing artifactual components from the dataset can then be formalized as a multiplication of *A* with *I*_*mod*_, where *I*_*mod*_ is a modified identity matrix that contains zeros for diagonal elements corresponding to the artifactual components to be removed:

$$\tilde{X}=AI_{mod}Y$$ (5)

Substituting (4) in (5) yields:

$$\tilde{X}=AI_{mod}A^{-1}X$$ (6)

in analogy to (1) we can thus define *W*_*ica*_ as:

$$W_{ica}=AI_{mod}A^{-1}$$ (7)

Similarly transformation matrices *W* can be derived for other approaches to decompose the data into artifactual and neurophysiological components, such as e.g. signal-space projection (SSP) [25,26], spatio-spectral decomposition (SSD) [27], signal space separation (SSS) [28], second order blind identification (SOBI) [29], blind source separation based on canonical correlation analysis (BSS-CCA) [30], and principal component analysis (PCA) [31,32].

#### Source analysis

Following linear transformations of the sensor level data, source analysis is a common analysis step. This raises the question if, for the source analysis, the previous modification of the data needs to be accounted for in the leadfield. The starting point of any source analysis is the following relation:

X=LQ (8)

Where *Q* is a matrix with source activity of various neuronal and artifactual sources over time (sources x time points) and *L* is a matrix with the physical forward models (leadfields) that describe the relation between neuronal and artifactual sources and the measured sensor signals (channels x sources). In general, the number of sources is arbitrarily high reflecting a continuous source distribution. It should be noted that, for other cases than the general considerations here, neuronal and artifactual data are often represented in separate terms of the equation.

#### Source analysis following linear sensor level transformations

Applying a linear transformation to the sensor level data (e.g. re-referencing or ICA cleaning, as described above) corresponds to a multiplication of (8) with the linear transformation matrix *W* from the left side, which yields:

$$\tilde{X}=WX=WLQ$$ (9)

For source analysis, we have to express the three-factor product in (9) as a two-factor product. There are two possibilities. Either the matrix *W* can be joined with the leadfield *L*:

$$\tilde{X}=\tilde{L}Q\text{, with}\;\tilde{L}=WL\;\text{being the modified leadfield}$$ (10)

or *W* can be joined with the source activity *Q*:

$$\tilde{X}=L\tilde{Q}\text{, with}\;\tilde{Q}=L^{-1}WLQ\;\text{being the modified source activity}$$ (11)

Unlike the modified leadfield $\tilde{L}$, the modified source activity $\tilde{Q}$ cannot be explicated algebraically unless strict constraints are imposed on *L* (*L* is normally rank deficient and thus constraints have to be imposed to find *L* ^*-1*^, such as e.g. *L* ^*-1*^ is a matrix with the property *L* ^*-1*^ *L = I, without requiring that L*L ^*-1*^ = *I*, where *I* is the identity matrix). However, the fact that *L* ^*-1*^ is practically difficult or even impossible to derive does not hinder the interpretation of the two alternatives:

From (10) and (11), it is evident that $\tilde{X}$ can be expressed as the product of the unmodified leadfield *L* and modified sources $\tilde{Q}$ or as the product of a modified leadfield $\tilde{L}$ and the original sources *Q*. Both alternatives are formally correct. To choose between the two alternatives, we need to consider their physical meaning and ask which sources we are aiming for. If we aim for the original sources *Q*, we need to work with (10) and employ a modified leadfield that compensates for the changes in the representation of the sensor level data. This is e.g. the case for re-referencing. However, the scenario is different for ICA-based cleaning. In this case, the aim is not to estimate the original *Q*, but to estimate a modified $\tilde{Q}$, i.e. *Q* without the artifactual sources. Thus, in this case, we need to work with (11), i.e. with the unmodified leadfield *L*.

In the following we formally show that both alternatives indeed yield the desired source activity.

To derive Q from (10) we need to solve the inverse problem given the modified data $\tilde{X}$ and the modified leadfield $\tilde{L}$. This is the very problem of source analysis, which is in general difficult to solve [14]. However, if successful, the original source distribution *Q* is recovered as aimed for in the first scenario.

The matter is more complicated for the second scenario. Using source analysis with the modified data $\tilde{X}$ and the original leadfield *L* results in $\tilde{Q}$. But is $\tilde{Q}$ the desired source activity? Substituting (7) in (11) yields:

$$\tilde{Q}=L^{-1}AI_{\mathrm{mod}}A^{-1}LQ$$ (12)

Then, substituting (8) and (4) in (12) yields:

$$\tilde{Q}=L^{-1}AI_{\mathrm{mod}}Y$$ (13)

*S = L* ^*-1*^ *A* can be conceptualized as a matrix with the source distributions of the independent components (sources x components). Again, the inverse of *L*, i.e. *L* ^*-1*^, is in general difficult to find, but this does not hinder the interpretation. Substituting this definition into (13) yields:

$$\tilde{Q}=SI_{\mathrm{mod}}Y$$ (14)

From this, we can explicitly see that, as intended, $\tilde{Q}$ consists of all but the sources of the artifactual components that are removed by zeroing the corresponding diagonal elements in *I*_*mod*_.

### 3. Effect of correcting the leadfield by mistake

We showed that the leadfield must not be corrected if linear transforms are applied for ICA-based artifact cleaning. But, what are the consequences if this is done by mistake? This question is important not only to judge the practical relevance of the present considerations for future studies, but also to assess previous studies that have applied a correction by mistake.

Wrongly correcting the leadfield, corresponds to applying (10) instead of (11). In other words, we do not consider the cleaned data $\tilde{X}$ to be due to cleaned sources, but due to the original sources—including the artifact—measured with modified sensors described by modified leadfields $\tilde{L}$ that are insensitive to the artifactual sources. What happens in detail if source-analysis is performed using this wrongly modified leadfield depends on the degree of artifact cleaning (i.e. number of removed components / variance) and the specific source analysis method applied. Nevertheless, the following considerations provide general insights.

Consider why we apply artifact cleaning before source analysis in the first place. The reason is that the artifactual sources ‘overlap’ with neuronal activity of interest. That is, artifactual sources and sources of interest share spatial characteristics that prevent a full separation at the source level. Removing artifactual components from the data thus increases the signal to noise ratio of the neuronal sources of interest.

This overlap between artifactual and neuronal sources of interest also determines the effect of modifying the leadfield by mistake. Measuring with a set of virtual sensors that are blind to the artifactual sources translates into a reduced sensitivity also for neuronal sources of interest that have a high overlap with the artifactual sources. Thus, the signal-to-noise ratio that is increased by removing artifactual sources from the dataset is subsequently reduced again by wrongly modifying the leadfield for source analysis. In other words, modifying the leadfield by mistake will counteract artifact cleaning.

To test this hypothesized effect of wrongly correcting the leadfield, we investigated the impact of removing muscular artifacts using ICA on analyzing visually induced gamma-band activity at the cortical source level. Neck muscle activity spatially and spectrally overlaps with neuronal gamma band activity [8,33–36]. Thus, visual gamma band activity provides a good test for the hypothesized effect of wrongly correcting the leadfield. We analyzed EEG data of 20 healthy subjects performing a perceptual decision task on a visual stimulus consisting of two high-contrast moving bars (see Material and Methods). We employed ICA to remove artifactual muscle components (number of rejected components: 38 ± 10.5, mean ± SD; 16–250 Hz band) and used beamforming to investigate neuronal activity resolved in time and frequency at sources within the visual cortex.

In accordance with previous reports [8,18,36–38], visual stimulation induced an increase of gamma power relative to pre-stimulus baseline in visual cortex (Fig. 1, blue line; two-sided t-test, p = 7.67 * 10^-5^, interval: 0.25–1.25 s). Removing artifactual ICA components that captured muscle activity significantly increased this gamma band response (Fig. 1, green line; two-sided t-test for ICA-cleaned response vs. non-cleaned response, p = 0.027, interval: 0.25–1.25 s). In other words, if we consider the gamma-band response as the ‘signal’ relative to the background ‘noise’, the ICA cleaning procedure increased the signal-to-noise ratio. This is because the gamma-band activity reconstructed in visual cortex did not only reflect the neuronal activity of interest, but, because of the proximity of neck muscles, also reflected artifactual muscle activity.

Next, we investigated the effect of wrongly modifying the leadfield before source analysis. Indeed, this led to a significant decrease of the visual gamma band response that even significantly dropped below the response for the raw data (Fig. 1, red line; two-sided t-tests for leadfield-corrected ICA cleaned response vs. non-cleaned and ICA-cleaned responses, both p < 4 * 10^-4^, interval: 0.25–1.25 s). Thus, this example accords well with the hypothesis that wrongly modifying the leadfield decreases the signal-to-noise ratio counteracting the benefit of ICA cleaning.

## Discussion

We showed that, depending on the physical meaning of the linear transformation applied to sensor level data, the leadfield must or must not be adapted before subsequent source analysis. For linear transformations that are applied to change the sensor level representation of the data, but not to remove sources from the measured data, the leadfield must be adapted. In contrast, for linear transformations that remove artifactual sources from the data, the leadfield must not be adapted.

We discussed ICA-based cleaning as an example for transformations that do not require a leadfield adaptation. However, the above arguments generally also hold for other approaches employed for artifact cleaning including signal-space projection (SSP) [25,26], spatio-spectral decomposition (SSD) [27], signal space separation (SSS) [28], second order blind identification (SOBI) [29], blind source separation base on canonical correlation analysis (BSS-CCA) [30], and principal component analysis (PCA) [31,32]. Furthermore, the above considerations also hold if, instead of removing artifactual components, only a few or one signal component of interest is selected for further analysis.

In our considerations, we assumed that artifactual ICA components captured only artifactual signals. In real-world applications this may not be the case. Artifactual components may also capture some neuronal activity that is then removed along with the artifact. This false removal of the signal of interest will naturally affect source analysis counteracting the improved SNR gained by removing artifacts, and may lead to miss-localization of neuronal activity. The specific consequence of such false removal of neuronal signal depends on the specific case and on the source analysis technique applied. However, in general, the problem will increase with the ratio of removed neuronal signal to removed artifactual signal. This advocates for a conservative approach when declaring components as artifactual. E.g. in an event related experimental design, one may demand that components declared as artifactual do not show any trask-related modulations. The problem of removing neuronal data, may be particularly relevant for signal-space projection (SSP) [25,26]. In signal space projection the data is projected away from a known artifact topography, while not specifically aiming a separation of neuronal and artifactual signals that would counteract removal of the signals of interest. Depending on the similarity of the artifact and neuronal topographies this can lead to severe distortions of subsequent source estimates [15]. Thus for source analysis following artifact cleaning using signal-space projection particular care is advised.

The provided framework also applies to scenarios, in which multiple linear transformations are applied in succession. For example, if ICA-based cleaning is followed by re-referencing at the sensor level, the leadfield must be adapted for the second, but not for the first transformation.

The effect of modifying the leadfield by mistake is of practical relevance to evaluate previous studies. While this effect depends on the relation of the applied modification to the sources of interest and the specific source analysis method employed, our considerations provide general insights that we verified in a real-world example. In short, wrongly modifying the leadfield will counteract the benefits of ICA cleaning. In the discussed example, this effect was so strong that ICA cleaning with a wrongly modified leadfield even resulted in a worse signal-to-noise ratio than no ICA cleaning at all. This loss in sensitivity should be considered when interpreting previous studies.

In general, future studies should carefully consider whether and, if so, how the leadfield must be changed for source analyses following linear transforms of the data.
