# Correction: Accounting for Linear Transformations of EEG and MEG Data in Source Analysis

**DOI:** 10.1371/journal.pone.0128689

**Published:** 2015-05-14

**Authors:** The PLOS ONE Staff

## Notice of Republication

This article was republished on May 1, 2015, to correct Fig 1, which was distorted during the production process. The publisher apologizes for the error. Please download this article again to view the correct version.
